# Does medical humanities matter? The challenge of COVID-19

**DOI:** 10.1136/medhum-2022-012602

**Published:** 2023-06-02

**Authors:** Jane Macnaughton

**Affiliations:** Institute for Medical Humanities, Durham University, Durham, UK

**Keywords:** medical humanities, health policy, arts in health/arts and health, medical anthropology, COVID-19

## Abstract

Medical humanities has tended first and foremost to be associated with the ways in which the arts and humanities help us to understand health. However, this is not the only or necessarily the primary aim of our field. What the COVID-19 pandemic has revealed above all is what the field of *critical* medical humanities has insisted on: the deep entanglement of social, cultural, historical life with the biomedical. The pandemic has been a time for reinstating the power of expertise of a particular kind, focusing on epidemiology, scientific modelling of potential outcomes and vaccine development. All of this delivered by science at speed.

It has been challenging for medical humanities researchers to find purchase in these debates with insights from our more contemplative, ‘slow research’ approaches. However, as the height of the crisis passes, our field might now be coming into its own. The pandemic, as well as being productive of scientific expertise, also demonstrated clearly the meaning of culture: that it is not a static entity, but is produced and evolves through interaction and relationship. Taking a longer view, we can see the emergence of a certain ‘COVID-19 culture’ characterised by entanglements between expert knowledge, social media, the economy, educational progress, risk to health services and people in their socio-economic, political ethnic and religious/spiritual contexts. It is the role of medical humanities to pay attention to those interactions and to examine how they play out in the human experience and potential impact of the pandemic. However, to survive and grow in significance within the field of healthcare research, we need to engage not just to comment. There is a need for medical humanities scholars to assert our expertise in interdisciplinary research, fully engaged with experts by experience, and to work proactively with funders to demonstrate our value.

## Introduction

The COVID-19 pandemic has served to accentuate the value of certain health-related disciplines while others have faded into the background. Clinical fields such as frontline emergency care, respiratory and intensive care have been prominent, and lab-based disciplines responsible for vaccine and drug development have been feted for saving millions and enabling a return to normal lives for so many on the planet (Gilbert and Green 2021). Those working in these fields have been regarded as ‘heroes’ in the battle against COVID-19. In this fast-moving context where the urgent search for solutions and the power of science to come up with answers has been at the forefront of everyone’s minds, slower burn, critical and questioning fields such as the medical humanities have been left seeking a role. As we now move into a world largely (as I write) in recovery from the pandemic, this is the moment to reflect on what role intellectually as well as practically medical humanities might play in a post-COVID-19 future.

When the pandemic was at its height, there was much discussion of a ‘new normal’ reflecting on lessons learnt in relation to global inequalities in public health responses and vaccine distribution (Bedford, Berglof, and Sridhar 2020). Now that the crisis is past, and the world has turned to a new one related to global security,1 the urgency of this call for change has faded but has not entirely gone away. A number of scholars have thrown down the gauntlet to the field of medical humanities to play a part answering it. In a recent blog post Kirsten Ostherr, who is professor of English at Rice University and runs the medical humanities programme there, identifies key contributions the arts and humanities might make to the COVID-19 crisis (Ostherr 2020). One is to give historical and cultural context to the pandemic: ‘we must learn to construct meaningful narratives that link human behaviour to data about disease’. The second is more forward looking, helping us ‘to understand how our decisions about whose life matters will shape the future to come’. This, Ostherr argues, is a critical value of the arts and humanities because they place health within its wider social, economic, cultural, religious and ethnic context and, crucially, are able to ‘craft compelling stories’ that enable us to imagine how life might be in the future. Ostherr’s call is for a mobilisation of the translational humanities (McLellan 2021) in order to identify emergent problems (such as anti-Asian violence) through historical and sociological insights, and to engage directly with the ‘experts’ managing the pandemic to ensure they connect effectively to their anticipated audiences.

This is a persuasive thesis and is one that is reflected in a great deal of activity in online conferences and workshops that have been taking place in the arts and humanities in the wake of COVID-19. One such initiative is that by the British Academy (BA) called ‘SHAPE the Future’ (British Academy 2020). The SHAPE acronym (standing for Social Sciences, Humanities and the Arts for People and the Economy) is set against science, technology, engineering and maths. The BA initiative insists on the importance of these SHAPE disciplines to inform future policy. The Academy held a series of workshops which led to five key principles for policymakers and researchers. These included the importance of using a broad knowledge base in policy making, not just focussing on the medical, biological and physical; the importance of being responsive to local and historical contexts and a renewed focus on inequalities and inclusivity, sustainability and the environment in our post-COVID-19 planning (Morgan Jones, Abrams, and Lahiri 2020).

Like Ostherr, the BA emphasise the need for broadening the sources of knowledge that might inform a post-COVID-19 recovery, and a sense that awareness brings with it a responsiveness to local and cultural contexts, circumstances and needs. One problem with this approach, of course, is that it seems to sink the humanities into an attitude of defensiveness and self-justification: a cry of ‘we matter too in these challenging times!’ However, what we can confidently assert is that those engaged in managing this crisis would have benefited from a less siloed approach to the sources of knowledge that determined their actions during the pandemic. The writer John Michael Colón sees the answer as building a more diverse fundamental canon for the humanities that is part of the liberal education of every student (Colón 2022). That approach of ensuring minds are primed to deal with the complexities of global health may be one solution. It may address the knowledge needed but not necessarily the methodological approach. This is a bigger ambition to be proposed here. It is part of the existing identity of a critically engaged medical humanities, but needs articulating in practical terms. Medical humanities should be involved in imagining a post-COVID-19 future and in helping to build it.

## Characteristics of a critically engaged medical humanities

Medical humanities has tended first and foremost to be associated with the ways in which the arts and humanities help us to understand health. In as much as it has *intervened* in healthcare, traditionally medical humanities’ role has been in the education of practitioners (Brody 2011). This approach, while being of value, influences only the way in which clinicians practice while having no effect on what they actually do. Newer, more ambitious conceptions of medical humanities have been described in this journal. ‘Critical medical humanities’ reflects the leadership of my own Institute at Durham University, UK. In their 2015 editorial, my colleagues William Viney, Felicity Callard and Angela Woods describe an approach that takes seriously the fact that the knowledge and methods of the arts and humanities are not just an add on, or enhancement to clinical practice, but that they have serious things to say about the how ill-health is produced across a range of sites and scales, and, indeed, how to tackle it. Their call is that medical humanities should:

… intervene more explicitly in … questions [of] aetiology, pathogenesis, intervention and cure—rather than. leaving such questions largely to the domains of the life sciences and biomedicine. (Viney, Callard, and Woods 2015, 3)

The ‘critical’ in critical medical humanities is not, therefore, negative but aims at constructive engagement with current health research drawing on critical theory to question the status quo and also to establish a platform for understanding bodies and their environments in ways that escape the confines of clinical assumptions. For example, Sarah Atkinson and her colleagues employ critical theory to point out the assumption that contemporary forms of (public health) governmentality characterise the individual as an autonomous agent holding responsibility for his or her own health and well-being (Atkinson et al. 2015, 75).

Critiquing this view of ‘individualistic liberalism’ may lead us to think of health as ‘something that is produced through the relations between bodies rather than something that a body is or is not’ (Atkinson et al. 2015, 77) and to seek recovery and response through collective group action rather than loading responsibility on a single person.

Critical medical humanities, therefore, serves to open out new insights and ways of conceptualising health and, importantly, aims to work in tandem with other disciplines, including biomedicine, in order to find innovative ways of addressing ill-health. As I hope will be clear from my analysis, critique in the sense of careful scholarly attention to the complex, entangled societal and political contexts in which health and ill-health are produced, makes an important contribution. But Kirsten Ostherr’s call to mobilise the humanities in essential service to a better postpandemic world is a real challenge to critical medical humanities in its desire also to intervene. A response is dependent on the exercise of these key features of interdisciplinary critique, exploration of, and intervention in, experience in spaces beyond the clinical, as well as engagement in global public health debates and planning. As historian Molly Worthen writes:

the pandemic has made disciplinary boundaries blurry again. This is the moment for champions of the medical humanities to strike. (Worthen 2021)

The most challenging aspect of this ‘second wave’ of medical humanities is to make the call to interdisciplinary engagement across science, medicine and the humanities disciplines meaningful in relation to changing lives and health for the better. It seems clear that as far as health funding in the UK is concerned, with the very considerable exeption of the Wellcome Trust, medical humanities has not broken through as a ‘go to’ discipline that might help address public or global health challenges. Medical researchers tend to connect with social science disciplines such as health geography or medical anthropology in order to consider lived experience in connection with the biomedical. I suspect that this is partly because these disciplines have a clearer identiy in respect of their methods and knowledge-base than medical humanities, which is a relatively new field in terms of research and whose identity has always been the subject of debate. My purpose here is to explore the ways medical humanities can indeed demonstrably ‘strike’ (in Worthen’s conception) and provide some potential answers for a more nuanced approach to public health in the future. Our field already has some good examples of how we can rethink approaches to ill-health by transforming institutional thinking as I will go on to describe (McLusky 2022, 17). There are, however, some challenges in relation to how we as a field engage constructively with global public health.

## Medical humanities as interdisciplinary critique

What the COVID-19 pandemic has revealed above all is what the field of critical medical humanities has always insisted on: the deep entanglement of social, cultural, historical, spiritual life with the biomedical (Viney, Callard, and Woods 2015). At the height of the pandemic, one of the fascinating aspects of living through it (were it not so devastating) is the fact that historians and cultural commentators are watching these elements of our collective lived experience connect and disconnect in real time. It was a bit like watching history in fast forward and made us as academic researchers wary of commenting too quickly or with apparent authority. This is challenging but one of the advantages of an interdisciplinary approach is the ability to untangle some of these elements to examine how they connect. To illustrate I will focus on two key points of discussion during the COVID-19 pandemic: responses to the inequalities revealed by COVID-19 and the relationship between religious belief and vaccine hesitancy.

### Inequalities and the metaphor of breath

The experiential and allied emotional trajectory of the pandemic has taken different shapes across nations, but there have been some commonalities. Aslam and colleagues examined over 140 000 news headlines from major international news outlets from December 2019 to June 2020 and noted a preponderance of headlines in the negative sentiment category, with the most common negative words being ‘pandemic’, ‘trump’, ‘outbreak’ and ‘virus’ (Aslam et al. 2020). The word ‘fear’ also features prominently as a fear of ‘death’ but less so fear of the key symptom of COVID-19, which, in its most serious form, is breathlessness. This, or allied words such as ‘breath’, somewhat surprisingly do not feature among the negatively emotive words. From a medical humanities perspective, however, the idea of ‘breath’ as a critical function for life and as a metaphor for our times has been central during the period of the pandemic signalling the way in which we as humans are united and how we are different. At the outset of the pandemic, people comforted themselves with a sense of solidarity and a feeling that ‘we are all in this together’ (Guterres 2020) but this soon collapsed as it became clear that the virus was killing more people from black, Asian and minority ethnic (BAME) communities and areas of lower socio-economic disadvantage (Whitehead, Barr, and Taylor-Robinson 2020). These COVID-19-oriented concerns about breath gained metaphorical force in May 2020 when George Floyd, a black American man, died after a police officer in Minneapolis knelt on his neck compressing his trachea for almost 8 min. His final words, “I can’t breathe”, echoing those of another victim, Eric Garner, became a rallying call for the new antiracist movement, Black Lives Matter, which galvanised people across the globe to gather in protest marches, despite the COVID-19 threat.

Breath and breathlessness were at the heart of the *Life of Breath* project (Life of Breath 2015-2020), which, as I will discuss in more detail later, built on entangled themes of breath (with its existential and spiritual overtones), suffocation, postcolonial oppression, silencing and inequality to develop specific clinical interventions for people with breathlessness.2 Nowhere was the entanglement of these themes more stark than in India at the height of the pandemic where images of poverty-stricken people searching hospitals for oxygen cylinders to relieve the suffering of their breathless relatives were some of the most distressing of the whole COVID-19 crisis. As Nasmima Selim comments:

Breathing and dying in pandemic times are […] profoundly embedded in racial (and other forms of social) injustice, evidence in how marginalised communities are left without the necessary access to care, suffering from the structural impossibility of breath. (Selim 2022)

This ‘structural impossibility of breath’ as metaphor for suffocation and oppression and as actual physical suffering stimulated significant calls for change and pathways to a new, more equal and caring future. One such is the response of the prominent UK epidemiologist, Sir Michael Marmot whose 2020 report, *Build Back Fairer: the COVID-19 Marmot Review* (Marmot et al. 2020), proposes a way forward to address the searing health inequalities emphasised by the epidemic. Within the academy, the call for ‘decolonising the curriculum’ has become louder and more attention has been paid by the Global North to scholars with authoritative voices from the South. The Cameroon philosopher and theorist, Achille Mbembe, summarises the current mood accurately in his short essay, ‘The Universal Right to Breathe’ (Mbembe 2020). He catches the sense, also in the Marmot report and across lay and other media, of a need not just to return to normal but to shape a ‘new normal’. In his essay Mbembe concludes:

Before this virus, humanity was already threatened with suffocation. If war there must be, it cannot so much be against a specific virus as against everything that condemns the majority of humankind to a premature cessation of breathing, everything that fundamentally attacks the respiratory tract, everything that, in the long reign of capitalism, has constrained entire segments of the world’s population, entire races, to a difficult, panting breath and life of oppression. To come through this constriction would mean that we conceive of breathing beyond its purely biological aspect, and instead as that which we hold in-common, that which, by definition, eludes all calculation. By which I mean the universal right to breath.

These entanglements between social views—often led by as well as reflected in the media—clinical response and scholarly interpretation are apparent in the comparisons between the COVID-19 pandemic and historical influenza outbreaks, especially those of 1918–19 and also 1957 and 1968 (Honigsbaum 2020b). The trajectory of this relationship has been a constantly shifting one. In early February 2020, the US edition of the magazine *Wired,* which discusses future science, culture and technology, cautioned against the notion, expressed in a personal health and fitness magazine, that ‘the flu is a bigger threat to the US’ (Khamsi 2020). This was a view that was prevalent at that time before COVID-19 really took hold, expressed across a range of media outlets including the *Washington Post* on 1 February 2020. As early as one month later, *The Times* of London was reporting the view of Liverpool-based virologist, Callum Semple, that it was ‘time to abandon the myth that coronavirus is no worse than flu’ (McLaughlin 2020). In a further twist, as governments eventually tried to persuade the population that the pandemic was over, the comparison with influenza was again being invoked as we were encouraged “learn to live with COVID, in the same way we have learned to live with flu” (Javid 2022). The comparison with influenza outbreaks, as the most recent and deadly in the global north is understandable, as it aligns the frightening unknown (COVID-19) with something familiar and potentially more predictable.

As these examples illustrate, the culture of COVID-19 was being produced and reproduced across these entangled sites of understanding and interpretation. However, despite the loss of life and outrage at inequality, we learn from history that even the devastating 1918–19 influenza epidemic is ‘largely “forgotten” by the collectivity of society’ (Honigsbaum 2020a). It is easy for society to forget and move on, disregarding the desire for a ‘new normal’ no matter how urgent and important this felt at the time. It remains to be seen whether and how we will remember COVID-19. Remembering is important, but it seems more urgent that the problems the pandemic emphasised, and in some cases revealed, including stark health inequalities, should be addressed more effectively in the future. Medical humanities can demonstrably pick apart these cultural entanglements but critical medical humanites has a more ambitious agenda: one of intervention not just explanation. What, then, can our field do to address these problems?

### Vaccine hesitance, conspiracy theories and the religious right

Vaccine hesitancy and how it has been addressed during the pandemic is illustrative of the ways medical humanities’ engagement might have helped deliver a more nuanced approach to public health in the early stages of vaccine roll out in the UK. The fact that BAME and disadvantaged groups were particularly vulnerable to infection and indeed death (Whitehead, Taylor-Robinson, and Barr 2021) did not seem initially to inform the roll out of the vaccine programme. The programme started in early December 2020 but it was not until later January 2021 that the first alarm calls began to sound from general practitioners whose close relationships with their patients enabled them to understand their hesitancy. One GP reported 20%–30% did not attend for vaccination among the BAME community compared with 2%–3% in other groups (Haynes 2021). Among this community concerns reported were the speed with which the vaccine had been developed, anxieties about permanent side effects and religious concerns about whether the vaccine contained alcohol or porcine products (Haynes 2021).

Some advance warning had been available about this issue. The UK Royal Society for Public Health carried out a survey in early December 2020 to explore public attitudes to taking the vaccine. Disturbingly, they found that only 57% of BAME respondents said they would get the vaccine compared with 84% of people who identified as white (Royal Society for Public Health 2020). The report warned that if these findings were ‘not acted upon [they] may exacerbate pre-existing health inequalities’ (Royal Society for Public Health 2020). The question then arises as to why those rolling out the programme had to rush to alter approaches within populations of concern at a later date.

The answer may lie in the dominance of a biomedical approach that does not necessarily (with notable exceptions such as the Marmot review) take seriously the deep-rooted cultural issues and systems of belief that determine how people act in relation to health problems. Within a crisis situation like the COVID-19 pandemic, there is an urgent need for the kind of courageous and speedy actions and solutions that science has the potential to provide. It is not that scientists, policy makers and government leaders were unaware of cultural concerns, but it may be that they loomed less large in this emergency where a quick and effective solution to mounting deaths was needed. Medicine, especially in such contexts, works on the assumption that evidence drives action: clinicians are guided by this and patients are assumed to respond appropriately. The COVID-19 vaccine has high efficacy and the implication is that we should all accept it as the most effective way out of the pandemic. But there are proximal and distal reasons why certain groups might not respond in this way. Most articles in the clinical and public health press did acknowledge the proximal, up-front issues such as lack of trust, safety concerns and problems of access (Razai et al. 2021). Addressing these problems required practical, immediate solutions, such as mobilising local knowledge, personal connections and trusted community champions and faith leaders to emphasise safety and demonstrate their own commitment to the vaccine (Razai et al. 2021).

Other, more deep-seated, hidden and distal issues are more difficult to address and to identify in the first place. It may be possible to address the specific problem of lack of trust by bringing in local leaders, faith leaders and champions, but this is an approach that will not counter the long-standing and deep-seated problem of structural racism that, intersecting with socio-economic disadvantage, has been the underlying cause of differential COVID-19 susceptibility as well as hesitancy (Paul, Fancourt, and Razai 2022). The assumption that all communities trust authority and will respond ‘like us’—that is, the included, white, middle class and by implication ‘reasonable’ citizen—may well demonstrate structural racism in action. Far from acknowledging and building this understanding into the vaccine programme, a controversial UK report published during the pandemic appeared to deny the existence of structural racism concluding that inequalities associated with COVID-19 were entirely owing to socio-economic disadvantage (Commission on Race and Ethnic Disparities 2021).

In the USA, vaccine hesitancy has also been a function of what has become known as Christian Nationalism. The online journal *The Conversation* reported in April 2021 that white evangelicals in the USA were the religious group least likely to say they would be vaccinated against COVID-19 (Barlow 2021). Nearly half said they would not get the jab against 30% in the general population. This again illustrates the way in which COVID-19 has become entangled with a developing cultural tendency to distrust science, medicine, expertise more generally and what might be seen as an institutional ‘elite’.

This lack of trust in institutions is problematic. The vaccine programme operates on the assumption that individuals are willing to take action in the present to prevent a future undesired event from happening, that is, infection with COVID-19. The anthropologist, Vincanne Adams has gone so far as to suggest that this ‘anticipatory episteme’ has become a ‘defining quality of our current moment’ (Adams, Murphy, and Clarke 2009, 246). Writing before the pandemic, Adams suggested that this ‘defining quality […] is its characteristic state of anticipation, of thinking and living toward the future’ (Adams, Murphy, and Clarke 2009, 246). At that time, this insight was directed towards things like risky health behaviours that might lead to problems in later life, but it is wholly appropriate for our current context. As Adams and colleagues say:

Anticipation is not just betting on the future; it is a moral economy in which the future sets the conditions of possibility for action in the present, in which the future is inhabited in the present. (Adams, Murphy, and Clarke 2009, 249)

However, history influences how people differentially experience the ‘current moment’ with consequences on how they might act. The experience of the pandemic has been profoundly different for individuals and communities variously exposed to the economic, emotional, spiritual and physical threats offered by the virus. This has been intense for many with countries in and out of lockdown with implications for additional childcare, loss of employment and economic austerity for already vulnerable groups. The assumption that individuals are willing to take the kind of anticipatory action required by the vaccine programme may not take into account the experience of people and communities whose lives may be characterised less by an orientation to the future but by living day to day with immediate concerns about job precarity, access to food or the current cost of living crisis. In addition, the vaccine programme asks people to consider our own individual ‘good’ in having a jab and the wider benefit to the population. Vaccine hesitancy has been described as a ‘threat’ to the success of the programme in suppressing the virus nationally and, ultimately, internationally (Larson and Broniatowski 2021). Such language, although often expressed in respectful terms, may also serve to exacerbate a ‘them and us’ culture already ingrained through historical othering of ethnic and religious groups and compounded by more immediate accusations of ‘selfishness’ and ‘stupidity’ (Duffy et al. 2020). Such an atmosphere of division might not necessarily lend itself to actions in favour of a wider good.

Taking into account perspectives that are not governed by the tenants of evidence-based medicine or presumptions about the ‘sameness’ of people’s lived experience is crucial for uncovering these deep-seated reasons for vaccine hesitancy. Public health approaches to vaccine uptake have tended to be on the backfoot in responding because of the complex, entangled, historical, religious and cultural background to hesitancy. As I have discussed, critical medical humanities can help uncover the hidden nature of this problem, and our approach, taking as our starting point the lived experience of communities and people, might contribute to rebuilding trust in healthcare institutions.

In their recent book on COVID-19 and shame, Cooper and colleagues comment that

The sciences alone […] may not value or create the kinds of outcomes cognisant of different forms of social, cultural and emotional survival or living well. Without a substantial—and genuinely receptive—engagement with the humanities, policy makers will continue to ask the wrong questions and look for answers in the wrong places. (Cooper, Dolezal and, and Rose 2023, 14)

This is a bold claim and aligns with the ‘critique’ identity of critical medical humanities I have discussed. It is, however, impossible not to respect and admire the heroic efforts of the ‘vaxxers’ who can justly claim to have saved millions of lives through the extraordinary speed with which they developed the COVID-19 vaccine (Gilbert and Green 2021). The challenge for medical humanities in getting involved in policy and public health spaces is to try to get engaged in developments as they happen rather than commenting in retrospect. Humanities scholarship tends to be slow burn and perhaps we need to be bolder and less fearful or getting it wrong; and to have the courage to change our narratives and intepretations as events unfold.

## Imagining and building a future: the role of the medical humanities

I hope it is clear so far that my argument has been that the medical humanities can disentangle the elements that make up our culture—specifically healthcare culture—and be aware of their several powers to influence how individuals and groups may act in relation major crises such as COVID-19. I have also suggested that the COVID-19 pandemic demonstrates clearly the meaning of culture: that it is not a static entity but is produced and evolves through interaction and relationship. As the critic Terry Eagleton reminds us, one of the original meanings of the word ‘culture’ was ‘husbandry’ or the tending of natural growth (Eagleton 2000, 1). This suggests (says Eagleton) both ‘regulation and spontaneous growth’:

The cultural is what we can change, but the stuff to be altered has its own autonomous existence, which then lends it something of the recalcitrance of nature. But culture is also a matter of following rules, and this too involves an interplay of the regulated and unregulated. (Eagleton 2000, 4)

Medical humanities critique can shake up and destabilise the certainties of medical culture through calling on the knowledge, methods and creativity of the humanities, social sciences and the arts. This shaking up is not intended to be destructive but constructive of creating new cultures that enable people to thrive in challenging circumstances and able to imagine more flourishing futures. One of the ways medical humanities does this is by asking questions from a different viewpoint or starting point from traditional biomedicine. The starting point is often driven by individual researchers’ passion for particular activist agendas in health which commit them to engage with activist or self-help groups who are trying to shift the structures, agendas and powerbases that govern what happens in healthcare. It is a clear sign of the health of this field that it is continually developing and during the 20 years I have led a medical humanities research institute a key change I have observed has been the emergence of a group of new researchers who are less discipline-based but rather identify themselves with an issue, whether that be anxiety, neurodiversity or medically unexplained symptoms.3 This often passionate engagement combines deep scholarship with a strong desire to make change happen for the communities and individuals affected.

To illustrate this in more detail, I will draw on two examples from our work at the Institute for Medical Humanities (IMH) at Durham in which we worked collaboratively with self-help groups taking responsibility for their own health.

### Managing intrusive voices

The *Hearing the Voice* project is a major interdisciplinary project based in IMH and funded over 10 years by the Wellcome Trust (Hearing the Voice 2010-2020). This project has been ground breaking in taking seriously the idea that hearing a voice in the absence of any speaker (regarded as a psychiatric symptom by the medical profession) can be part of normal human experience and is especially common after bereavement for example, or in religious or spiritual experience. Working closely with the Hearing Voices Network (HVN), a national charity supporting people who hear voices, the project has engaged with voice hearers who reject the dominant medical discourse around their experiences and seek to explore and express their own management and recovery narratives. They are the ‘experts by experience’ on their voices and assert the right to make sense of them in relation to that experience, to find their own meanings and shared meanings within the network. With this turn to a collective search for meaning and agency, my colleague Adam Powell argues, voice-hearers necessarily reject existing systems while formulating new ones, undertaking what anthropologist Claude Lévi-Strauss would have termed a kind of ‘conversion’, in which an individual having been deemed a patient emerges as one cured, or at least in control of their own cure (Powell 2018). The success of this process of conversion lies in the ‘collectively validated articulation of the problem’ by the group—not in the empirically validated solution of medicine (Powell 2018, 16). Our medical humanities approach supported this validation by untangling the individual and collective experience from the confines of conventional medical dominance: supporting the creation of a new culture that does not reject the benefits of a clinical approach but enables this to co-exist with a group inspired culture that respects powerful individual stories of empowerment and recovery (Woods, Hart, and Spandler 2022).

### Managing breathlessness: re-envisioning the body through dance

As part of the *Life of Breath* programme,2 we undertook a project that illustrates how medical humanities can overturn perceptions of the body in chronic illness and open out new possibilities for those with chronic breathlessness.

A key challenge in managing chronic breathlessness is poor uptake of the major medically evidence-based treatment: pulmonary rehabilitation (PR). PR involves participants in undertaking exercise in a gym-like space to strengthen their breathing muscles and improve lung function and exercise capacity. Engaging in exercise is always challenging, but much more so for people whose breathing is problematic. Our interdisciplinary work on *Life of Breath*, involving anthropologists, literary scholars, philosophers working with clinicians and people with experience, provided further insights into the problems of language. As part of a series of meetings called ‘Breath Labs’, we gathered together people with experience, clinicians and the project academics to discuss specific themes in relation to breathlessness, one of which was language. The patients did not like the implications of ‘rehabilitation’, which they felt further stigmatised their already marginalised condition, and the gym was not a space that felt familiar culturally to them (Harrison et al. 2020). Key also to our thinking was our work with clinical neuroscientists on embodied awareness among people with breathlessness. It appeared that part of the reason why there might be a mismatch between experience of breathlessness and the results of formal clinical assessment was that people with breathlessness have reduced interoceptive awareness, interoception being the sensation of our bodily functions, such as breathing, or the movement of bones and muscles (Macnaughton 2020). Discussions in our research group with a colleague involved in managing a breathlessness forum in London, who also happened to be taking a course in dance, started us thinking about the value of dance movement in addressing some of these issues.

Dance might be more culturally familiar, and its language was not stigmatising and most of all, it might give people whose bodies had become a burden a feeling that there was pleasure to be had in engaging with their bodies, and a sense that the body might again be beautiful in movement. We opened this suggestion out to a local British Lung Foundation ‘Breathe Easy’ support group and worked with them and a local dance teacher (supported by our dance colleague from London) on a series of specially developed dance movement classes. The outcome satisfied the requirements of clinical symptom improvement, showing improved exercise capacity, strength and balance among participants, while their perception of breathlessness was low (Harrison et al. 2020, 6). Key to the success of the programme, however, lay in the bonding of the group, of learning and being together and a common commitment to staying well. The group spontaneously produced a wordle diagram in which the keywords were ‘friendship’, ‘fun’, ‘laugh’ and ‘inspire’ (Harrison et al. 2020, 4).

Research on this project demonstrated for me three key insights that I think are crucial to underline the potential transformative effect of a very different approach to managing a public health problem. First, it was an artist, not a doctor or other clinician, who lead the dance ‘treatment’ programme. The importance of this was that artists regard people not as patients, as problems to be solved, but as people with creative potential—to learn, to develop new understanding or new ways of moving and enjoying the body. Second, the process of addressing the problem was, like the HVN example, owned by the group. They had agency in the solution; it was not just something ‘done’ to them, but invested in by them, through time, effort and (eventually) belief. With their own health at stake, the group instinctively rejected an individualised, singular and bounded conception of the body to one that recognised that bodies do well together in connection (Atkinson et al. 2015, 77).

Third and connected to this important group experience, these interactions reminded us that lived experience is a dynamic thing, and human beings are not the static entities seen in the clinic as a problem needing solving by clinical input alone. They need to be conceptualised as in constant conversation with their social, cultural and environmental surroundings. As in the example of the HVN, people in the breathlessness group found new shared meaning in a group activity that liberated them from the old static certainties of respiratory decline into a sense that they could take charge of their own—if not recovery—but healthier and happier lifestyle. Anthropologist Tim Ingold strongly asserts this dynamism in his book *Biosocial Becomings* (Ingold and Palsson 2013). He reflects on the kind of ‘impasse’ created by regarding human beings through a traditional institutional medical culture: a machine-like way initiated at the Enlightenment, and suggests we think differently:

to think of ourselves not as *beings* but as *becomings*—that is not as discrete and pre-formed entities but as trajectories of movement and growth. (Ingold and Palsson 2013, 8)

He goes on:

Life is a task, and it is one in which we have, perpetually, never-endingly and collaboratively, to be creating ourselves. (Ingold and Palsson 2013, 8)

From these examples it is clear that medical humanities, engaging with the range of cultures that produce and support both health and illness, and challenging institutional rules and assumptions, has the power to transform ways of improving health by focussing on the problem and on the potential for growth and development that human becomings possess, even in the context of illness that is (clinically) incurable.

## Conclusion: challenges for our field

The projects above do not relate directly to the challenge of COVID-19 but I hope are illustrative of what a medical humanities approach can achieve. However, these examples do not address the question of how medical humanities can *itself* transform in the service of a post-COVID-19 world and in particular to be part of addressing the significant public health challenges present in that world. A major challenge for public health-oriented medical humanities is that our field has tended to focus on the experience of the individual rather than the collective. As Lise Saffran notes, ‘population health approaches the subject of human experience from the aggregate, rather than the individual’ (Saffran 2014, 106). Saffran’s suggestion for how to overcome this difference of focus is to use narrative practices to bring public health and epidemiological data alive and relevant to policy makers, government and the public. I think this approach has power but it rather suggests a retrospective role for our field. We need to go further and be fully present in shaping policy in the first place.

I have suggested ways in which engagement between public health and medical humanities might have helped address some of the challenges of vaccine hesitancy and painted a picture of how small-scale projects at a local level can transform assumptions about illness and recovery. I have shown that through its critically engaged approach medical humanities has the power to suggest and to carry through programmes of change. What our field has not achieved, however, is a voice within the spaces and places that have large-scale responsibility for change. Medical humanities remains peripheral, at best adjunctive to policy and practice in health and medicine.

Prior to the pandemic, a call was led by Julia Kristeva to launch a ‘global think tank’ on medical humanities to deconstruct the ‘difference between hard and soft science’ and build a new view of ‘evidence’ that challenges the dominance of biology (Kristeva et al. 2018, 57). More recently, during the pandemic, a new field of ‘planetary health humanities’ was suggested as the means of ensuring wider conceptions of health and well-being achieved their rightful place (Lewis 2021). These calls, reaching back as they do to the old ‘Two Culture’ debates about the hegemonic power of specific disciplines (Snow 1998) seem unlikely to succeed. Overcoming the barriers to progressing medical humanities research within public health, and more generally, is challenging, but identifying these barriers and thinking through potential solutions is a first step, as outlined in a recent report by the IMH (McLusky 2022). To conclude, I will pick out two major issues, aware that these do not provide answers but potential ways forward.

Medical humanities is in many ways defined by *how* we undertake research rather than *what* we actually investigate. We need confidently to assert our skills in interdisciplinary collaboration. No other field within health and medicine has such a wide reach across disciplines and experience in making interdisciplinarity work. In September 2016, the UK Academy of Medical Sciences published an ambitious report entitled *Improving the Health of the Public by 2040*, which acknowledged that

biomedical science … does not have the capacity to address [the] increasingly diverse and complex issues that transcend disciplinary, sectoral and geographical boundaries. (Academy of Medical Sciences 2016, 5)

The Academy specifically called on the arts and humanities to engage in collaborative research and in helping to prepare the next generation of researchers with the skills to face this complexity. As an educator as well as an interdisciplinary researcher, I have been advocating at my home institution for a module that all students would undertake focussing on a global challenge, such as climate change, pandemics or global mental health, which would enable students from across disciplines to come together to work collaboratively on a related project. It seems to me that those of us in higher education fail if we do not prepare our students for the complexities of understanding a problem and of addressing it collaboratively. Related to this, we need to recognise the importance of interdisciplinary publication and embrace the additional work involved in demonstrating our successes to publics within clinical medicine, health policy and the humanities. This is time consuming and requires nuancing of approaches to publication, as well as learning how to express project successes as ideas that may be translatable to other health fields.

Following on from this to my second point: ours is a relatively new field especially in research terms and we have work to do on our visibility and ability to influence. I was delighted, for example, that the Academy of Medical Sciences report strongly asserted the role of medical humanities in public health but was disappointed that the authors had not engaged with any experts in our field in the UK. Clearly, no one is going to do this for us: our field has a responsibility to expand the ambition of research within the academy and to forge the connections to make this possible. Medical and public health funders are not banging at our doors asking for us to engage; as a new research field we need to lobby funders, explain our relevance and demonstrate that through our exemplary and award-winning projects (McLusky 2022, 17). In the process, medical humanities can be a beacon for change in a research culture that has become toxic, in which research success is measured by quantifiable outputs, and where the success of research process is ignored in favour of outcomes. In a recent report by the Wellcome Trust, aspects of positive research culture have been identified as collaboration, enabling participation, valuing diversity and inclusion, interdisciplinarity in approach and respecting all contributions (Wellcome Trust 2020). If we were to write a manifesto for critical medical humanities research, these would be the keywords. In the context of the current need to transform research cultures, making research more enjoyable and fruitful for scholars and publics alike, medical humanities has a major opportunity to demonstrate its value and step into the mainstream.

## Data Availability

No data are available.

## Notes

1. In February 2022, Russia invaded Ukraine and at the time of writing this conflict is causing significant concern in relation to global security, food and energy supplies.

2. The *Life of Breath* was a research programme funded by Wellcome from 2015 to 2020 exploring the experience of breathlessness: https://lifeofbreath.webspace.durham.ac.uk/ (accessed 26 August 2022).

3. I menton these issues as foci of some of the researchers I work with in the Institute for Medical Humanities at Durham University.
